# Nanoscale reorganization of sarcoplasmic reticulum in pressure-overload cardiac hypertrophy visualized by dSTORM

**DOI:** 10.1038/s41598-019-44331-y

**Published:** 2019-05-27

**Authors:** Sina Hadipour-Lakmehsari, Amine Driouchi, Shin-Haw Lee, Uros Kuzmanov, Neal I. Callaghan, Scott P. Heximer, Craig A. Simmons, Christopher M. Yip, Anthony O. Gramolini

**Affiliations:** 1 Translational Biology and Engineering Program, Ted Rogers Centre for Heart Research, Toronto, Ontario M5G 1M1 Canada; 2 Department of Physiology, Faculty of Medicine, University of Toronto, Toronto, Ontario M5S 1M8 Canada; 3 Department of Biochemistry, Faculty of Medicine, University of Toronto, Toronto, Ontario M5S 1A8 Canada; 4 Institute of Biomaterials and Biomedical Engineering, University of Toronto, Toronto, Ontario M5S 3G9 Canada; 5 Terrence Donnelly Centre for Cellular and Biomolecular Research, University of Toronto, Toronto, Ontario M5S 3E1 Canada

**Keywords:** Cardiac hypertrophy, Super-resolution microscopy

## Abstract

Pathological cardiac hypertrophy is a debilitating condition characterized by deleterious thickening of the myocardium, dysregulated Ca^2+^ signaling within cardiomyocytes, and contractile dysfunction. Importantly, the nanoscale organization, localization, and patterns of expression of critical Ca^2+^ handling regulators including dihydropyridine receptor (DHPR), ryanodine receptor 2 (RyR2), phospholamban (PLN), and sarco/endoplasmic reticulum Ca^2+^-ATPase 2A (SERCA2A) remain poorly understood, especially during pathological hypertrophy disease progression. In the current study, we induced cardiac pathological hypertrophy via transverse aortic constriction (TAC) on 8-week-old CD1 mice, followed by isolation of cardiac ventricular myocytes. dSTORM super-resolution imaging was then used to visualize proteins at nanoscale resolution at two time points and we quantified changes in protein cluster properties using Voronoi tessellation and 2D Fast Fourier Transform analyses. We showed a decrease in the density of DHPR and RyR2 clusters with pressure-overload cardiac hypertrophy and an increase in the density of SERCA2A protein clusters. PLN protein clusters decreased in density in 2-week TAC but returned to sham levels by 4-week TAC. Furthermore, 2D-FFT analysis revealed changes in molecular organization during pathological hypertrophy, with DHPR and RyR2 becoming dispersed while both SERCA2A and PLN sequestered into dense clusters. Our work reveals molecular adaptations that occur in critical SR proteins at a single molecule during pressure overload-induced cardiomyopathy. Nanoscale alterations in protein localization and patterns of expression of crucial SR proteins within the cardiomyocyte provided insights into the pathogenesis of cardiac hypertrophy, and specific evidence that cardiomyocytes undergo significant structural remodeling during the progression of pathological hypertrophy.

## Introduction

The sarcoplasmic reticulum (SR) is a multi-functional organelle that is essential in the proper functioning of cardiomyocytes. The SR has many functions involved in maintaining normal cellular physiology, including protein synthesis, lipid metabolism, and Ca^2+^ cycling^1,2^. In cardiomyocytes, the SR mediates Ca^2+^ flux during Ca^2+^-induced Ca^2+^ release (CICR). During CICR, action potential-induced Ca^2+^ inflow through sarcolemmal L-type Ca^2+^ channels (e.g. dihydropyridine receptor or DHPR) triggers SR Ca^2+^ release from the ryanodine receptor 2 (RyR2) into the cytosol, resulting in myofiber contractions. The sarco(endo)plasmic reticulum calcium ATPase 2A (SERCA2A) and its inhibitor phospholamban (PLN) mediate SR reuptake of Ca^2+^ in advance of the next contractile cycle^3^. Ca^2+^ cycling within the cardiomyocyte is significantly altered in patients with heart disease. Changes in expression levels and biochemical properties in many of the SR proteins, including DHPR, RyR2, SERCA2A, and PLN are well established^4–7^. In addition to these widescale biochemical changes, spatial changes in L-type Ca^2+^ channels and changes of microdomain interactions between potassium channels and caveolin-3 have been shown to trigger arrhythmias in heart failure^8,9^. In this paper, our focus was to investigate and characterize the nanoscale spatial changes of critical SR proteins in a murine model of pressure-overload cardiac hypertrophy.

Recently, advances in super resolution microscopy approaches have resulted in unparalleled visualization of proteins with near single-molecule resolution^10,11^. Such advances maintain the high specificity of antibody-based confocal microscopy, while achieving greater spatial resolution. This study aims to characterize the reorganization of the SR proteins in isolated pressure-overload murine adult cardiomyocytes. Using direct stochastic optical reconstruction microscopy (dSTORM), we visualized the changes in localization, clustering, and patterns of expression of several proteins critical in SR Ca^2+^ cycling in hypertrophic hearts. These approaches provide novel characterization of the physiology underlying the pressure-overload cardiac hypertrophy phenotype and provides new mechanistic insights into this disease. Here, we used dSTORM imaging to determine SR protein cluster properties and regional organization in a murine model of pressure-overload cardiac hypertrophy via transverse aortic constriction (TAC). We have shown a decrease in DHPR and RyR2 protein cluster density and an increase in PLN and SERCA2A protein cluster density with hypertrophy. We have also demonstrated a trend towards greater dispersion and disorganization in DHPR and RyR2 with TAC. This is in contrast to the greater sequestration observed in SERCA2A and PLN with hypertrophic cardiac disease. Our findings reveal a robust reorganization in SR protein clusters in nanoscale in response to pathological cardiac hypertrophy.

## Materials and Methods

### Experimental animals

All animal work was conducted under the guidelines of the Canadian Council on Animal Care. As previously described^12^, TAC was applied to the descending aorta by placing a 7-0 silk suture with the aid of a 27-gauge hypodermic needle on CD1 mice (Charles River Laboratory) at 8 to 10 weeks of age. Sham mice underwent the same surgical procedures except the ligature was not tightened; surgical details have been published previously^12,13^. Hearts were harvested 2 weeks and 4 weeks post TAC or sham surgery and cardiomyocytes were isolated.

### Adult mouse ventricular cardiomyocytes isolation

All experimental procedures were conducted in accordance with the Animal Care Guidelines approved by University of Toronto Animal Use and Care Committee. Protocol for isolation of adult mouse cardiomyocytes was modified from Ackers-Johnson *et al*.^14^. Briefly, Sham-, 2-week TAC-, and 4-week TAC-operated CD1 mice hearts were perfused with EDTA buffer and perfusion buffer before being digested with warmed collagenase II buffer (250 units/mL; LS004176, Worthington Biochemical Corp., Lakewood, NJ, USA). Following dissociation and washing, 3 stages of sedimentation were performed to remove the non-myocyte fraction before being suspended in culture media and plated on laminin-coated glass-bottom dishes. Cells were fixed 2 hours after plating and processed for immunofluorescence experiments.

### Immunoblotting

Protein lysates from mouse heart tissues were harvested in radioimmunoprecipitation assay buffer (RIPA, 50 mM Tris-HCl; pH7.4, 1% NP-40, 0.5% sodium deoxycholate, 0.1% SDS, 150 mM NaCl, 2 mM EDTA), supplemented with protease and phosphatase inhibitors (Roche), for 30 mins on ice, spun down at 15,000 × *g* at 4 **°**C. Soluble fractions were saved for immunoblotting. Protein lysates were heated for 5 min to either 65 °C or to boiling, run on 4–12% polyacrylamide gels and transferred onto 0.22–0.45 μm nitrocellulose membranes. After blocking for 1 hour with 5% milk in 0.05% TBS-Tween20, primary antibodies were added and incubated at 4 **°**C overnight: primary mouse monoclonal anti-DHPR (1:500 dilution; ab2864; Abcam), primary mouse monoclonal anti-RyR2 (1:1000 dilution; ab2827; Abcam), primary mouse monoclonal anti-PLN (1:1000 dilution; MA3-922; ThermoFisher), primary mouse monoclonal anti-SERCA2A (1:1000 dilution; MA3-919; ThermoFisher), and primary rabbit polyclonal anti-NCX1 (1:1000 dilution; ab151608; Abcam).

### Immunofluorescence and confocal microscopy

Cardiomyocytes were plated on glass-bottom dishes (MatTek Corp., Ashland, MA, USA) for immunofluorescence and confocal imaging. For immunofluorescence analysis, isolated cardiomyocytes were fixed with 4% paraformaldehyde (PFA) for 30 minutes at 4 degrees Celsius. Next, cardiomyocytes were incubated with permeabilization buffer (0.5% Triton X-100, 0.2% Tween-20 in PBS) for 30 minutes at 4 degrees Celsius. Blocking buffer (5% FBS in permeabilization buffer) was then added and incubated for 30 minutes at room temperature. Primary antibodies (listed above) were then added (SERCA2A – 1:500, PLN – 1:1000, RyR2 – 1:1000, DHPR – 1:700). Cardiomyocytes were then incubated with primary antibody overnight at 4**°**C and fluorophore-conjugated secondary antibody staining (Alexa 647, Molecular Probes) was performed at room temperature for 1 hour in the dark. Nuclear counterstaining was performed using 1 μg/ml Hoechst 33342 (Cell Signaling, #4082) at room temperature for 15 minutes in the dark.

### TIRF microscopy

TIRF microscopy was performed on a home-built TIRF microscopy system integrated with an Olympus FluoView 500 confocal microscope using an IX-70 base (Olympus, Canada) using a high numerical aperture 60x oil-immersion objective (NA = 1.45, Olympus, Japan). A thin layer of index-matching oil (n = 1.518) was used to couple the objective optically to the glass surface of glass-bottom dishes (MatTek Corp. Model 155409, Ashland, MA, USA). Excitation of Hoechst was achieved using an analog modulated 405 nm diode laser (Power Technology, Model LDCU12/6516). Excitation of AF647 was achieved using an analog modulated 643 nm laser (Power technology, Model LDCU5/A109) with a maximum measured power of 90 mW at the source and 20 mW at the objective during dSTORM experiments. A clean-up notch filter (ZET642/20x, Chroma, Bellow Falls, VT) was used to clean the excitation spectrally. Fluorescent images were captured using a water-cooled eXcelon-equipped Evolve 512 EMCCD camera (Photometrics, AZ, USA) using µ-Manager (version 1.4.19).

### dSTORM imaging and processing

To initiate stochastic photoswitching for dSTORM, a photoswitching buffer was added prior to imaging. This buffer consisted of 50 mM cysteamine (2-mercaptoethylamine, Sigma-Aldrich), 40 µg/ml catalase (Catalase, from bovine liver, aqueous solution, Sigma-Aldrich), 0.5 mg/ml glucose oxidase (from *Aspergillus niger*, Sigma-Aldrich), 50% w/v glucose (D-glucose, Sigma-Aldrich) diluted in PBS, pH 7.4 and provided conditions that yielded a high photon count for AF647. This buffer modulates the photophysical properties of AF647 by scavenging oxygen and creating a reducing environment. A 643 nm laser was set to a power of 20 mW measured after the objective and was used to drive AF647 into an off-state prior to having a sparse subset of fluorophores coming back on in a stochastic manner over the acquisition period. To reconstruct a super-resolved image, 10000 images over a period of 300 s were acquired each with an exposure of 30 ms. Images were processed using the ImageJ plugin ThunderSTORM (version 1.3) with the linear least square (LLS) localization parameter. Following localization and reconstruction, the coordinates of single emitters were filtered based on their localization precision (uncertainty value) and photon count in order to discard electronic noise (0 nm < localization precision <7 nm) and sample noise (localization precision >60 nm). Despite the care taken in processing blinks, the obtained super-resolved localization coordinates do not offer an absolute measurement (*i*.*e*. the ability to count the number of proteins in a region) of protein count but a relative, quantitative representation of protein distribution and clustering.

### Cluster analysis

Cluster analysis was conducted using Voronoi Tessellation via the SR-Tesseller software (http://www.iins.u-bordeaux.fr/team-sibarita-SR-Tesseler)^15^. Upon generation of Voronoi polygons, the cell object was defined by manually thresholding for points that have mean distances outside of the cell boundary. Clusters are defined as objects with a density factor of 2, a minimum area of 2 and a minimum number of localization of 50. We chose 50 as a minimum based on the assumption that each protein was tagged by multiple secondary antibodies each carrying approximately 5 fluorophores, as estimated by the antibody manufacturer. In addition, for labeling and photophysical consistency, we have used identical Alexa Fluor 647-conjugated secondary antibodies for all four proteins. Cluster characteristics were exported and statistical analysis was performed on cluster area, number of localizations per cluster and cluster density.

### Periodicity analysis

Regions of interest (ROIs) ranging between 10 × 10 to 20 × 20 μm were selected from dSTORM rendered images for each protein for Sham, 2 week-TAC and 4 week-TAC (10 ROIs from 10 cells per protein per condition per n). Sarcomeres were oriented vertically using the Fiji rotate function and specifying the rotation angle determined from the fast Fourier transform (FFT). Upon generation of the FFT for each ROI, power spectra are averaged per protein per condition and a small region of the power spectra containing higher frequencies was selected (w: 1.13, h: 0.60, x: 9.68, y: 9.94) and individual frequency plots were generated. The minimum baseline floor was subtracted from each plot and the area under the curve (AUC) was calculated as a metric for the presence or absence of a clear periodicity and the degree of 2D organization. One-way ANOVA statistical analysis was then applied on the AUCs.

## Results and Discussion

### Tunicamycin-induced cell stress leads to differential SR protein dynamics

**Figure 1:**
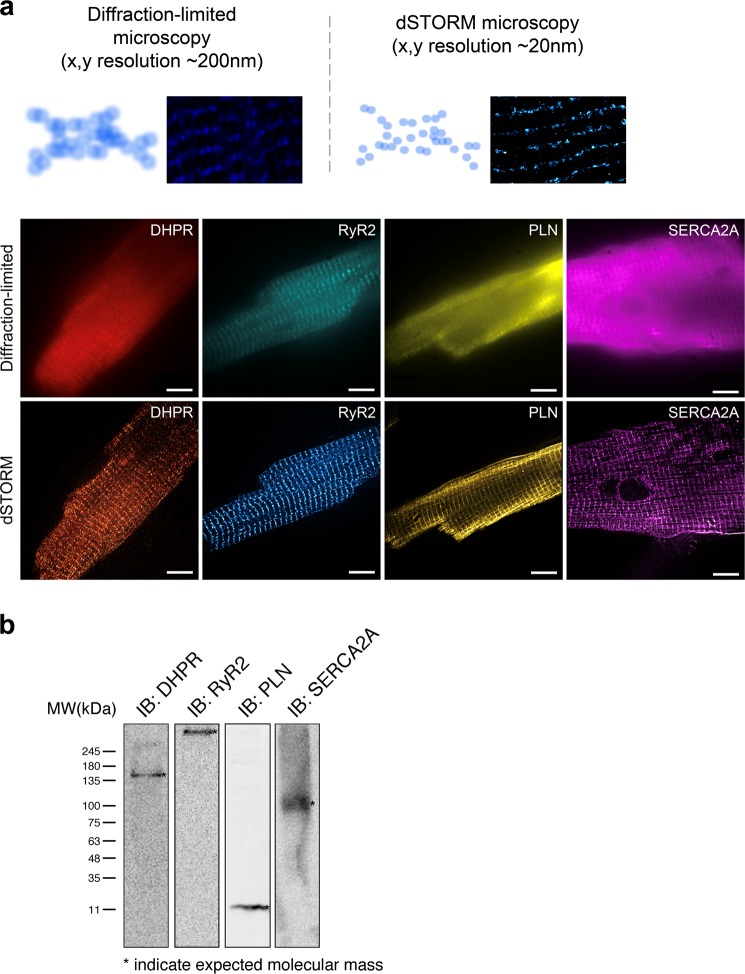
Schematic comparison of diffraction-limited microscopy and true nanoscale imaging via dSTORM. (**a**) Schematic diagram demonstrates the difference in spatial resolution between diffraction-limited microscopy and dSTORM imaging. A comparison between diffraction-limited microscopy and dSTORM imaging of our isolated adult mouse ventricular myocytes stained for DHPR, RyR2, PLN, and SERCA2A shows true molecular scale visualization of protein cluster properties. (**b**) Immunoblot analysis of DHPR, RyR2, PLN, and SERCA2A in isolated adult cardiomyocytes show the detection of their expected molecular weight bands.

Changes in protein spatial distribution and organization are often accompanied by significant alterations in the downstream signaling pathways that are difficult to capture using diffraction-limited microscopy and hence, are often overlooked. Traditional diffraction-limited microscopy can only achieve a lateral resolution of 150 nm while super resolution imaging via dSTORM can achieve a lateral resolution of 30 nm, enabling more accurate visualization of proteins and provide better localization of individual fluorescent reporters. In addition, since dSTORM generates sets of coordinates per capture, several cluster analysis methods can be leveraged to analyze these datasets. A comparison between diffraction-limited microscopy and dSTORM imaging of isolated adult mouse ventricular myocytes stained for DHPR, RyR2, PLN, and SERCA2A is shown in Fig. 1a. To confirm the specificity of the selected antibodies, immunoblot analysis of DHPR, RyR2, PLN, and SERCA2A in isolated adult cardiomyocytes was performed and showed no non-specific binding detected (Fig. 1b). Moreover, as a control for our immunofluorescence experiments, secondary antibody control studies were performed to further substantiate the findings of our study (Supp. 2).

**Figure 2:**
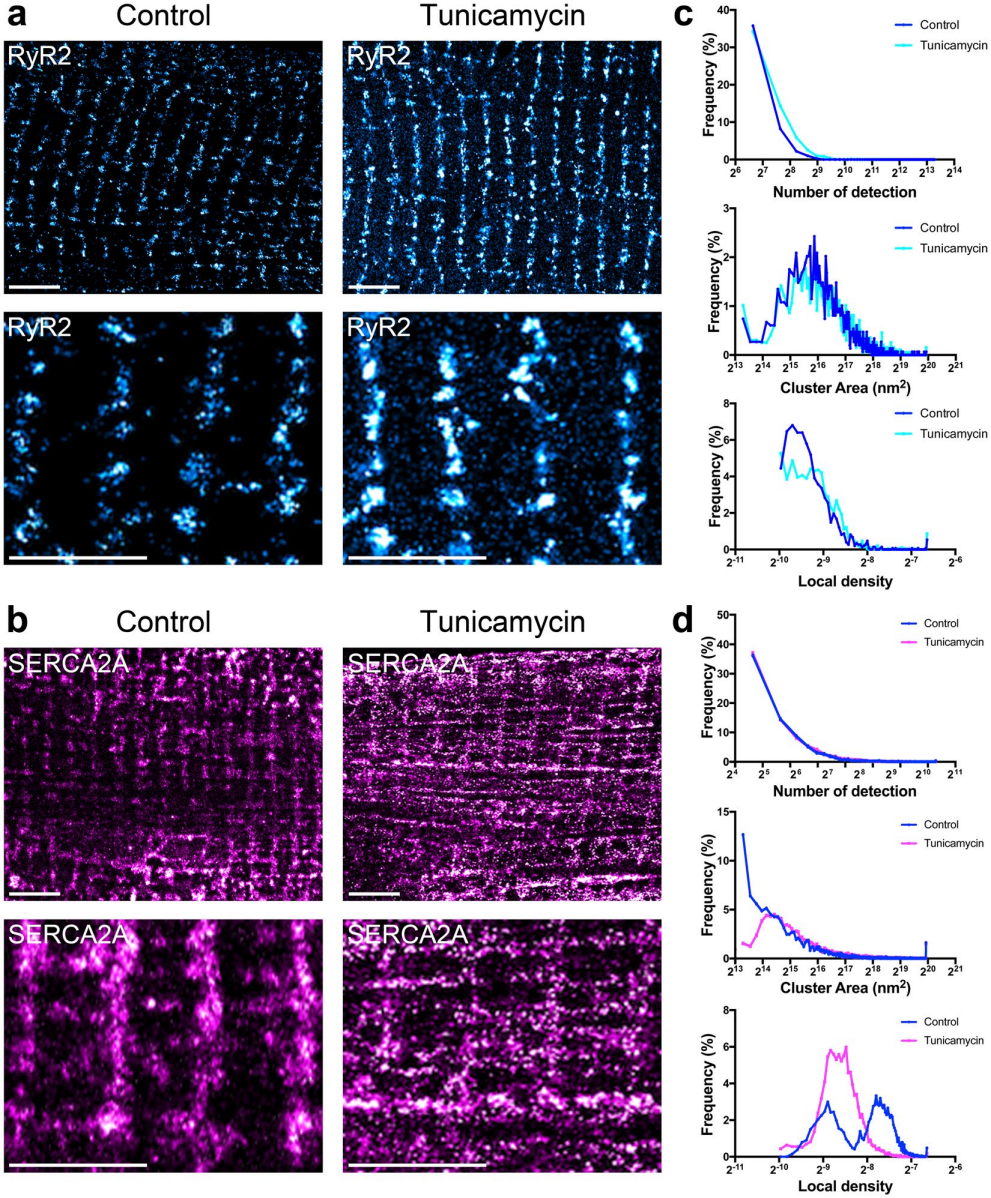
dSTORM imaging reveals cellular stress induces nanoscale changes in SR protein cluster properties. (**a**–**d**) Adult mouse ventricular cardiomyocytes were isolated, treated with control and 5 μg/mL tunicamycin conditions, and immunofluorescence was conducted to visualize (**a**) RyR2 and (**b**) SERCA2A by dSTORM imaging. (**c**,**d**) Voronoi tessellation was performed to quantify and plot changes in protein cluster properties, number of detection, cluster area, and cluster density (right). (**a**,**b**) Top panels scale bar = 3 μm, bottom panels scale bar = 2 μm. N = 10 cells per protein per condition.

As a proof of principle, we used dSTORM imaging to look for changes in the nanoscale organization of SR proteins in isolated adult mouse cardiomyocytes subjected to SR stress *in vitro*. Here, we used 3-hour tunicamycin (5 μg/mL) incubation as a SR stress inducer. As shown in Fig. 2a, incubation with tunicamycin led to a more dispersed and disorganized distribution of RyR2. Quantitative protein cluster analysis plotted as distribution curves also revealed changes in cluster properties, including an increased number of detections per cluster and larger cluster areas (Fig. 2c). Representative images for SERCA2A with and without cellular stress are shown in Fig. 2b. Following SR stress, SERCA2A, similar to RyR2, was disorganized and dispersed within the sarcomeres. Distribution curves also demonstrated changes in cluster properties, with a higher population of spatially larger and less dense SERCA2A clusters after tunicamycin treatment (Fig. 2d). These findings demonstrated the versatility of our dSTORM imaging and processing method and confirmed that under stress, SR proteins undergo significant reorganization in cardiomyocytes.

### Cardiac pressure overload leads to disorganized distribution of Ca^2+^ regulatory proteins

**Figure 3:**
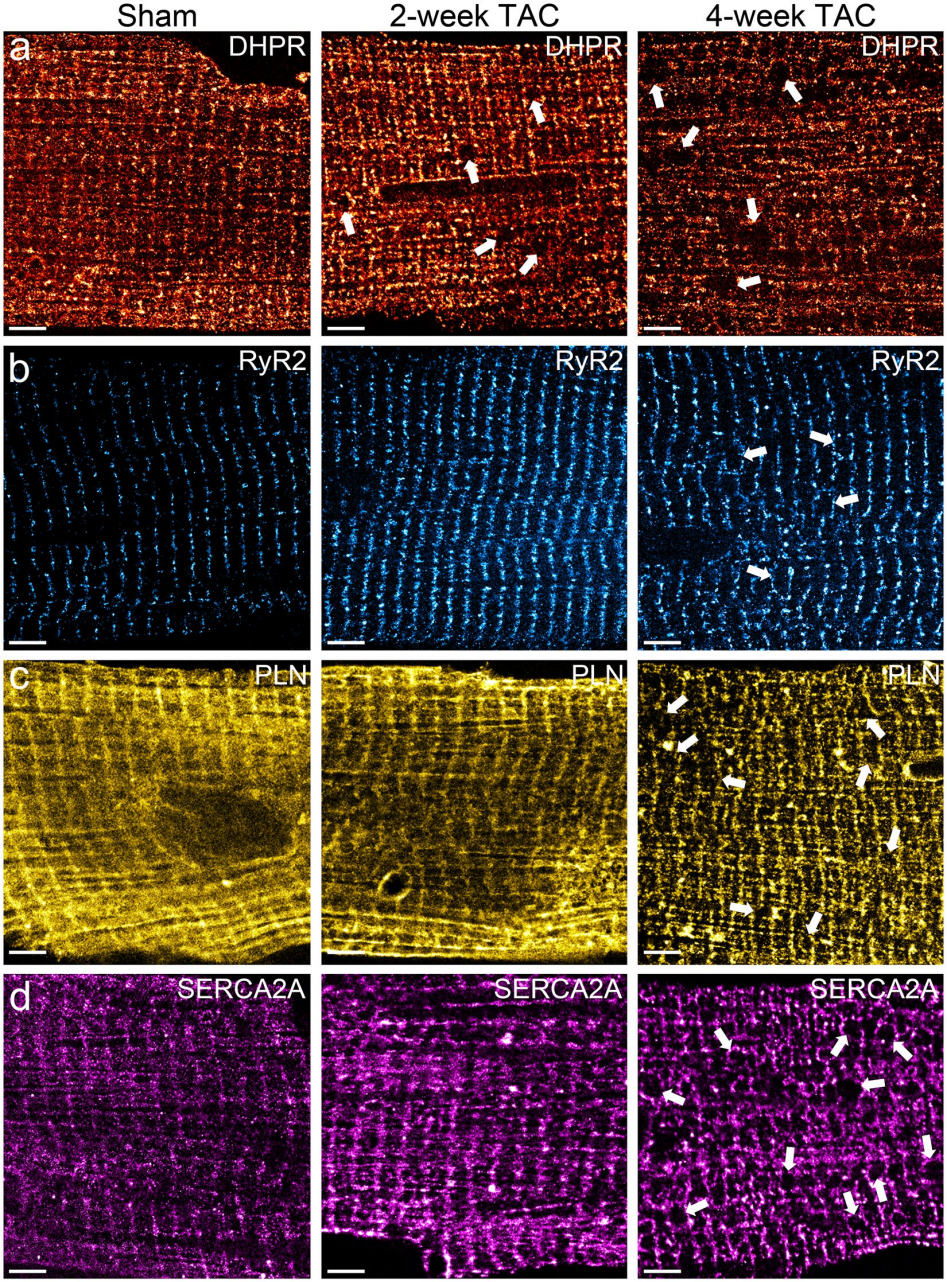
Pressure overload-induced cardiac hypertrophy leads to morphological changes in the localizations of major SR Ca^2+^ regulating proteins. (**a**–**d**) Representative immunofluorescence dSTORM images of adult cardiomyocytes isolated from sham, 2-week TAC, and 4-week TAC mice stained for (**a**) DHPR, (**b**) RyR2, (**c**) PLN, and (**d**) SERCA2A. The localization and expression patterns of these target proteins change dramatically in pressure-overload cardiac hypertrophy leading to a dispersion of DHPR and RyR2 and a sequestration of PLN and SERCA2A. Scale bar = 3 μm. Sample size: 10–12 cells per protein per condition, n = 3.

Next, we isolated ventricular cardiomyocytes from sham-operated, 2-week post-TAC, and 4-week post-TAC adult mice. Transverse aortic constriction surgery and time-point analysis was performed as previously described by Platt *et al*.^12^. Progressive pathological left ventricular hypertrophy was observed at 2 and 4-week post-TAC with peak left ventricular pressure observed at 4-week post-TAC surgery. However, little is known about the pathological alterations in molecular reorganization and pattern of expression of critical Ca^2+^ regulating proteins that occur in TAC. Here, we investigated the time course of protein cluster properties including cluster density, pattern expression, and pattern organization at a nanoscopic level to improve our understanding of the molecular alterations that occur in TAC. After immunofluorescence and dSTORM imaging, protein cluster properties and regional protein organization patterns were quantitatively measured. To characterize potential spatiotemporal changes of various important SR proteins, we qualitatively analyzed their patterns of expression in cardiomyocytes of 2-week and 4-week post-TAC hearts. As shown in Fig. 3, the normally well-organized structure of the protein clusters dissipates as indicated by the greater disarray of their organization. For DHPR, 2-week TAC and 4-week TAC led to the presence of large circular gaps (arrows) that disrupted the regular striated pattern of DHPR localization in control cells (Fig. 3a). In addition, there was widely observed disorganization and dispersion of labeled DHPR upon cardiac hypertrophy, leading to a significant loss of DHPR striations. For RyR2, the ordered localization of RyR2 became more diffuse with TAC as RyR2 localized within the empty spaces between striations (Fig. 3b). In contrast, the diffuse PLN and SERCA2A expression in sham cardiomyocytes changed to more sequestered and dense patterns of expression in TAC (Fig. 3c,d). Furthermore, large circular gaps became apparent in TAC cardiomyocytes immunolabelled for SERCA2A, similar to those seen in DHPR-labelled cardiomyocytes. While the SR proteins presented similar striated staining patterns, spinning-disk confocal analysis of NCX1 co-stained with DHPR, RyR2, PLN, and SERCA2A in adult cardiomyocytes as an internal control experiment showed distinctive subcellular localization to their respective subdomains within the muscle structure (Supp. 1a–c).

As discussed earlier, changes in expression levels and biochemical properties in many of the SR proteins, including DHPR, RyR2, SERCA2A and PLN have been well established. However, our data reinforces the emerging idea that nanoscale spatial changes of critical SR proteins in cardiac phenotypes may represent an additional concern.

### Transverse aortic constriction induces changes in the clustering properties of SR proteins

**Figure 4:**
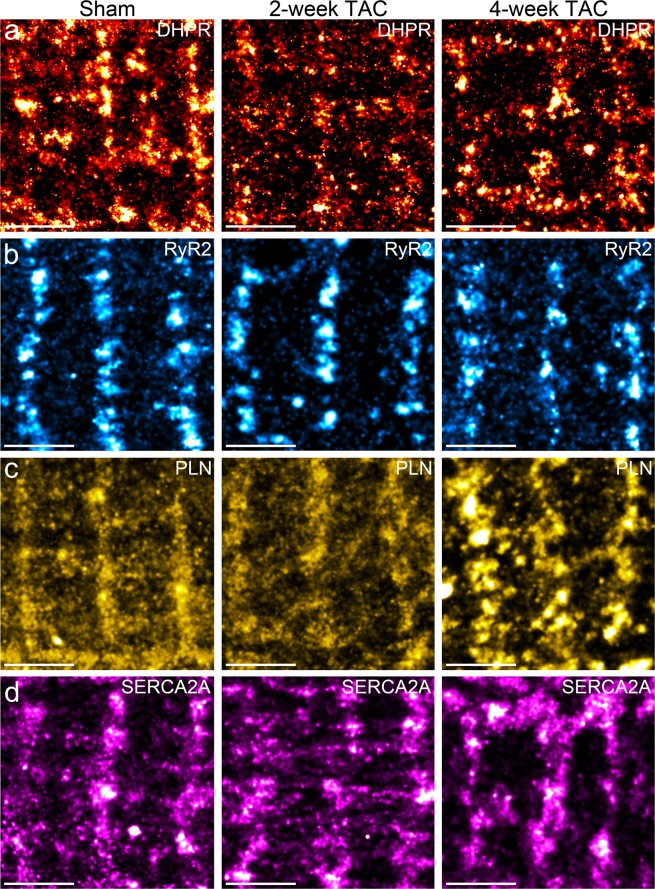
Pathological cardiac hypertrophy induces qualitative, visual differences in clustering properties of major SR Ca^2+^ regulating proteins. (**a**–**d**) Representative magnified regions of the dSTORM images reveal distinct, visual differences in the cluster properties of target SR proteins in adult mouse cardiomyocytes isolated from TAC pressure-overload hearts (**a**) DHPR, (**b**) RyR2, (**c**) PLN, and d) SERCA2A. Scale bar = 2 μm. Sample size: 10–12 cells per protein per condition, n = 3.

**Figure 5:**
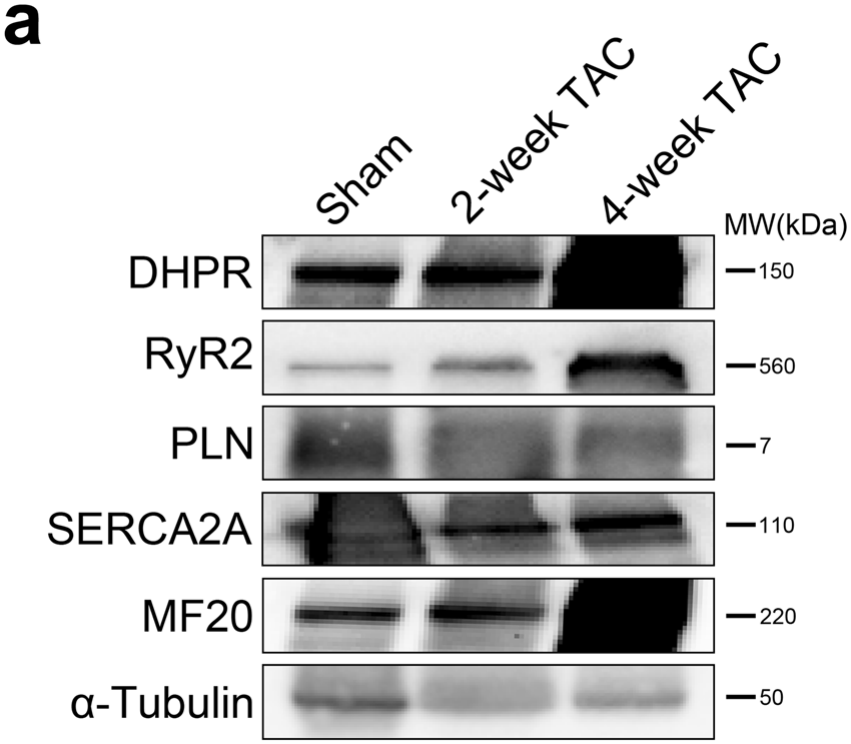
Pressure-overload induced hypertrophy lead to differences in protein expression levels of Ca^2+^ regulating SR proteins. (**a**) Immunoblotting analysis of DHPR, RyR2, PLN, SERCA2A, and MF20 (sarcomeric myosin) in sham, 2-week TAC, and 4-week TAC cardiomyocytes showed their respective protein expression levels at their respective molecular weight. α-tubulin was used as an internal control. Immunoblots were cropped to highlight the specific bands of interest, with the uncropped full-length blots presented in Supplementary Fig. 1.

Protein cluster analysis was conducted using Voronoi Tesselation on dSTORM datasets^15^. For this analysis, we analyzed the number of detections and the area per cluster. Cluster density represents a ratio of the number of detections and cluster area per given cluster. Representative protein clusters for each protein are shown in Fig. 4a–d. Here, we report changes in clustering characteristics for DHPR, PLN, SERCA2A and RyR2 in TAC hearts. The average values for these cluster properties have been displayed in Table 1. Similar as before, immunoblotting analysis of DHPR, RyR2, PLN, and SERCA2A showed increasing expression levels of DHPR and RyR2 in 2-week and 4-week TAC cardiomyocytes compared to sham-operated cardiomyocytes (Fig. 5a). In contrast, a significantly decreased expression level of monomeric PLN and SERCA2A was observed in 2-week and 4-week TAC cardiomyocytes compared to sham-operated cardiomyocytes (Fig. 5a). Additionally, immunoblot probed for myosin heavy chain β (MF20), a marker for cardiac hypertrophy^16,17^, showed low expression at 2-week TAC but were dramatically increased by 4-week TAC, confirming physiological compensation two weeks post TAC that progressed to pathological hypertrophy by 4 weeks post TAC (Fig. 5a).

**Table 1:** Average values of quantified cluster properties for DHPR, RyR2, PLN, and SERCA2A under sham, 2-week TAC, and 4-week TAC conditions.

		DHPR	RyR2	PLN	SERCA2A
	Sham	169 ± 2	243 ± 2	457 ± 31	262 ± 6
Number of Detection	2-week TAC	113 ± 6^*^	138 ± 2^*^	348 ± 8^*^	288 ± 6^*^
	4-week TAC	144 ± 3^†,‡^	212 ± 3^†,‡^	524 ± 32^‡^	403 ± 7^†,‡^
	Sham	89, 800 ± 4, 920	116,913 ± 2,950	121,056 ± 20,175	95,858 ± 2,438
Cluster Area (nm^2^)	2-week TAC	95, 605 ± 6, 484	126, 533 ± 2, 362	67, 708 ± 1, 621^*^	105, 680 ± 2, 665^*^
	4-week TAC	107, 016 ± 3, 712	114, 740 ± 1, 310^‡^	128, 760 ± 7, 951^‡^	130, 084 ± 2, 544^†,‡^
	Sham	0.00246 ± 1.638e-5	0.00259 ± 1.585e-5	0.00553 ± 4.001e-5	0.00301 ± 1.208e-5
Cluster Density (Number of detection/nm^2^)	2-week TAC	0.00157 ± 2.501e-5^*^	0.00147 ± 2.462e-5^*^	0.00641 ± 2.32e-5^*^	0.00312 ± 1.459e-5^*^
	4-week TAC	0.00163 ± 2.259e-5^†^	0.00213 ± 1.685e-5^†,‡^	0.00472 ± 1.32e-5^†,‡^	0.00367 ± 1.409e-5^†,‡^

*p < 0.05 sham vs 2-week, ^†^p < 0.05 sham vs 4-week, ^‡^p < 0.05 2-week vs 4-week.

**Figure 6:**
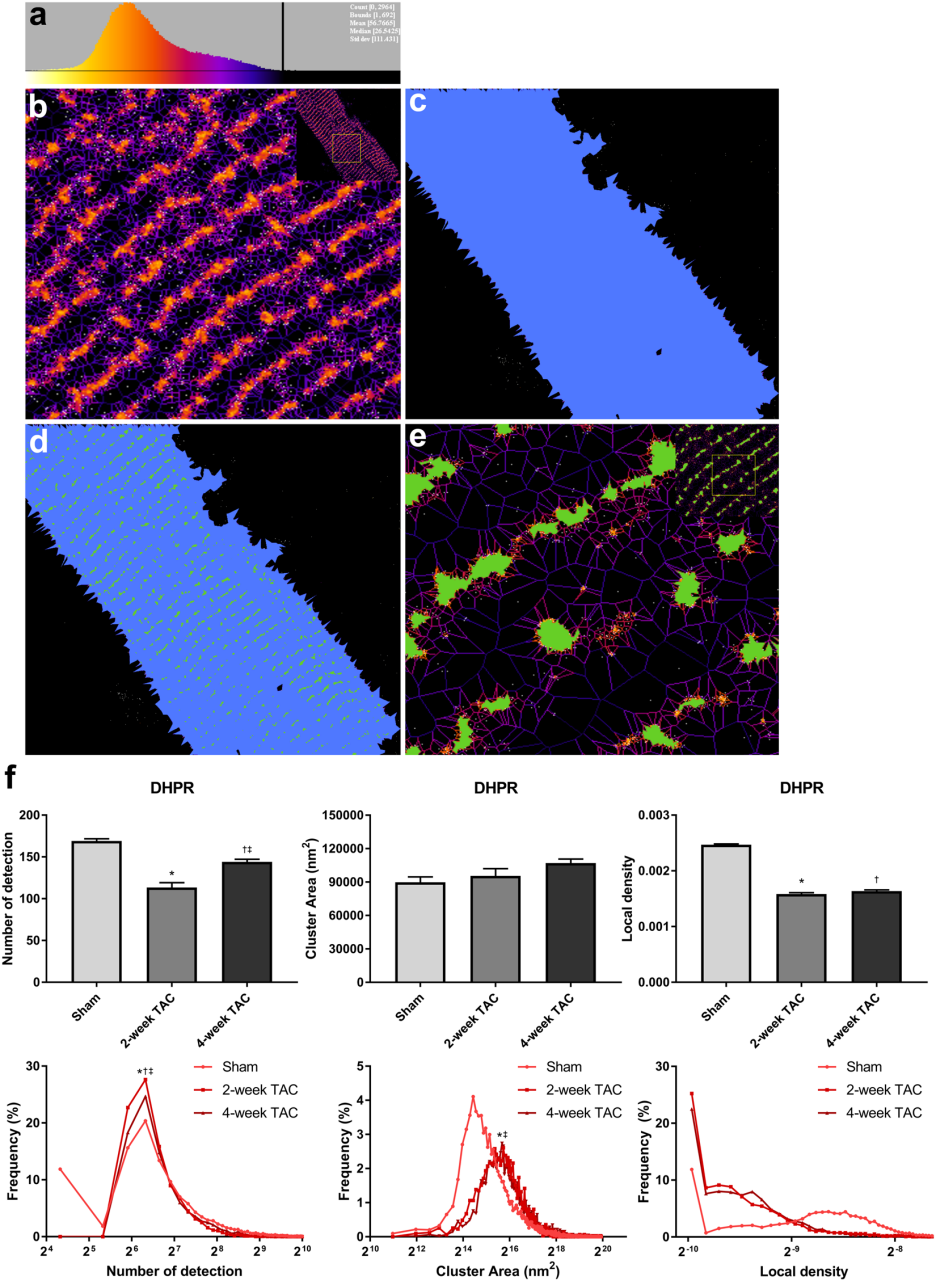
Voronoi tessellation analysis defines cluster properties for each identified cluster. (**a**) Mean distance distribution generated from Voronoi Tessellation is used to reject background localizations. Black line indicates upper limit of proteins being considered for analysis. (**b**) Upon background subtraction only localizations present within the cell boundary are considered for cluster analysis. (**c**) All localizations within the cell are contained within the cell object (blue). (**d**) Clusters containing at least 50 localizations (green) overlaid on cell object (blue). (**e**) Clusters overlaid on voronoi polygons to extract cluster area, cluster density, and number of detection for each detected cluster. (**f**) Voronoi tessellation analysis shows average and distribution curves for number of localizations, cluster area, and cluster density in sham, 2-week TAC, and 4-week TAC cardiomyocytes stained for DHPR. Sample size:10–12 cells per protein per condition, n = 3; *p < 0.05 sham vs 2-week, ^†^p < 0.05 sham vs 4-week, ^‡^p < 0.05 2-week vs 4-week.

Next, in order to provide a comprehensive view of the changes in cluster properties of our target SR proteins, we analyzed the average values for each measurement, as well as the distribution curves. This allowed us to not only observe overall changes, but to also discover changes in sub-populations of protein clusters. We have used SR-Tesseler to extract clustered regions with the aim of comparing cluster area, number of localizations per cluster, and cluster density. Localizations that fall outside of the cell boundary are rejected based on their mean distance distribution (Fig. 6a,b). All the localizations within the cell boundary are segmented to form the cell object (Fig. 6c). Clusters are defined by setting a minimum value for the number of fluorophores required for a set of points to be considered a cluster (Fig. 6d,e see details in Materials and Methods). Finally, we applied ANOVA statistical tests to the average values of the cluster properties and Mann-Whitney statistical tests for nonparametric distribution of the distribution curves.

DHPR, also known as Ca_v_1.2, is a voltage-sensitive Ca^2+^ channel located in the transverse tubules (t-tubules) that is responsible for Ca^2+^ intake into the cell upon stimulation, and thereby initiating the process of contraction. In our studies, the average number of DHPR localizations per cluster in hypertrophic cells decreased (Fig. 6f), going from an average of 169 ± 3 in sham cells to 113 ± 6 in 2-week TAC cells and 144 ± 3.2 in 4-week TAC cells. This decrease corresponded to a higher frequency (~28% in 2-week TAC and 25% in 4-week TAC) of clusters with a number of detection of 64. The average cluster area showed a modest increase in hypertrophic cells, which may be attributed to the greater population of large diffuse clusters, increasing in mean cluster area from 89,800 ± 4,920 nm^2^ in sham to 95,605 ± 6,484 nm^2^ in 2-week TAC and 107,016 ± 3,712 nm^2^ in 4-week TAC. Overall, these changes in cluster area and number of localizations were associated with a significant decrease in average local density. Specifically, the distribution curves for 2- and 4-week TAC cardiomyocytes showed an increase in the frequency of less dense clusters with density values of 0.001 number of detection/nm^2^. The average DHPR cluster density (number of detection/nm^2^) decreased from 0.0025 ± 1.6e-5 in sham cardiomyocytes to 0.0015 ± 2.5e-5 in 2-week TAC and 0.0016 ± 2.3e-5 in 4-week TAC (Fig. 6f).

**Figure 7:**
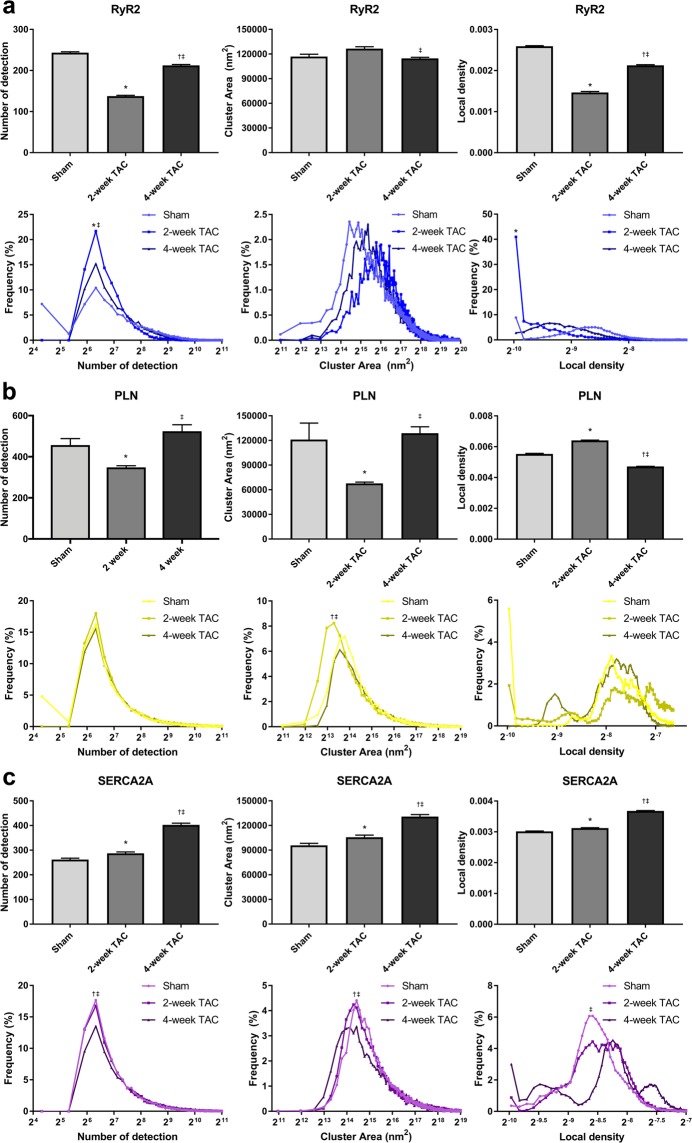
Quantitative Voronoi tessellation analysis yields distinct changes in protein cluster properties with TAC. (**a**–**c**) Average and distribution curves for number of localizations per protein cluster, cluster area, and cluster density in sham, 2-week, and 4-week TAC cardiomyocytes were determined via Voronoi tessellation and plotted for (**a**) RyR2, (**b**) PLN and (**c**) SERCA2A using a region of interest which encapsulated the whole cell. Sample size:10–12 cells per protein per condition, n = 3; *p < 0.05 sham vs 2-week, ^†^p < 0.05 sham vs 4-week, ^‡^p < 0.05 2-week vs 4-week.

RyR2 is an important SR protein responsible for Ca^2+^ release from the SR upon stimulation. Here, RyR2 showed a remarkable decrease in the average number of localizations per cluster in 2-week TAC cells and a more modest decrease in 4-week TAC cells, relative to sham (Fig. 7a). The average number of detections per cluster was approximately 243 ± 2 for sham cells, 138 ± 2 for 2-week TAC cells, and 212 ± 3 for 4-week TAC cells, corresponding to an increase in the incidence of smaller clusters, which contained between 64 and 128 localizations. Similarly, hypertrophic cells had a larger mean cluster area size, increasing from 116,913 ± 2,950 nm^2^ in sham to 126,533 ± 2,362 nm^2^ in 2-week TAC and normalizing to 114,740 ± 1,310 nm^2^ in 4-week TAC. As expected, there was a higher frequency of larger sized clusters in TAC cardiomyocytes as demonstrated by the rightward shift of the distribution curve. Lastly, the cluster density changed from 0.003 ± 1.585e-5 detections/nm^2^ in sham cardiomyocytes to 0.001 ± 2.5e-5 in 2-week TAC and 0.002 ± 1.7e-5 in 4-week TAC (Fig. 7a).

It would be interesting to speculate that such changes occur as a potential response of the myocyte to disperse its L-type Ca^2+^ channels in order to release Ca^2+^ in a more widespread manner in the cell, ultimately, in an attempt to increase its contractile efficiency during disease. Future studies could clarify whether this is simply a systemic pathological alteration in localization or is part of a more concerted compensatory response to maintain contractility under duress. There is considerable evidence that Ca^2+^ transients are extended in duration in TAC cardiomyocytes, and that altering DHPR and/or RyR2 can lead to a dyssynchronous CICR, ultimately, leading to various arrhythmias^18–21^. RyR2 is hyperphosphorylated in cardiac disease and heart failure, increasing its sensitivity and probability for Ca^2+^ release from the SR^4^. It has been shown that a smaller nearest-neighbour distance between RyR2 protein clusters can also substantially increase the potential for aberrant Ca^2+^ release^20^. Our data shows a dispersion of RyR2 clusters across the cardiomyocyte, thus reducing its nearest-neighbour-distance. These changes in Ca^2+^ cycling in disease would increase the probability for arrhythmias and may explain the presence of such arrhythmias in pressure-overload cardiac hypertrophy.

The SERCA2A inhibitory protein, PLN, is another important protein in the regulation of Ca^2+^ cycling in cardiomyocytes. Previous work has shown PLN to be hypo-phosphorylated in heart disease resulting in reduced SERCA2A function^22^. We investigated whether the pattern of expression of PLN was altered with TAC. The average number of detection for PLN decreased significantly from 457 ± 31 in sham to 348 ± 8 in 2-week TAC and recovers to 524 ± 32 in 4-week TAC (Fig. 7b). In the distribution curve, both 2-week and 4-week TAC cells lost the population of clusters with 16 localizations. In addition, 2-week TAC cells had a higher population of clusters with 64–128 localizations, which normalized to sham levels in 4-week TAC. Average cluster area for PLN had the same pattern as number of detection with a drop in 2-week TAC to 67,708 ± 1,621 nm^2^ from 121,056 ± 2,0175 nm^2^ in sham and a recovery to 128,760 ± 7,951 nm^2^ in 4-week TAC. The distribution curve revealed a leftward shift towards a greater population of smaller clusters. The greater increase in number of detection relative to the increase in cluster area led to an increase in the average local density of 2-week TAC compared to sham and 4-week TAC (0.0055 ± 4.0e-5 in sham, 0.0064 ± 2.3e-5 in 2-week TAC, and 0.0047 ± 1.3e-5 in 4-week TAC). The distribution curve demonstrated drastic changes in the density and revealed subpopulations that were unique in 2-week TAC and 4-week TAC cardiomyocytes (Fig. 7b).

The major protein responsible for Ca^2+^ sequestration following contraction is SERCA2A. We observed SERCA2A increased its average number of localizations per cluster from 261.7 ± 6.1 in sham cells to 287.5 ± 5.8 in 2-week TAC, and 402.7 ± 7.1 in 4-week TAC. This increase corresponded to a reduced frequency of clusters containing 64–128 localizations, especially in 4-week TAC cells (Fig. 7c). There was a slightly higher frequency of clusters containing greater than 256 localizations. SERCA2A showed a higher cluster area average rising from 95,858 ± 2,438 nm^2^ in sham to 130,084 ± 2,544 nm^2^ in 4-week TAC cells. The distribution curve showed a left-shift in the cluster area and should correspond to a lower average cluster area for SERCA2A; however, there was a greater frequency of subpopulations with larger cluster areas, thus increasing the average SERCA2A cluster area. With regards to local density, 2-week TAC cardiomyocytes had similar SERCA2A cluster density average values to sham cells (0.0031 ± 1.2e-5 and 0.0030 ± 1.5e-5 detections/nm^2^, respectively). However, due to a larger increase in the number of detection as opposed to cluster area, the average density of 4-week TAC cardiomyocytes was moderately increased (0.0036 ± 1.4e-5) (Fig. 7c). In the distribution curve however, the unimodal distribution of SERCA2A cluster density of sham cardiomyocytes becomes multi-modal in both 2-week and 4-week TAC cardiomyocytes. This data reveals various subpopulations of various densities with hypertrophic disease progression. The importance of the distribution curve again becomes apparent as it reveals information that would have been overlooked by averages of the different values.

### Cardiac hypertrophy leads to changes in the organization of SR proteins

**Figure 8:**
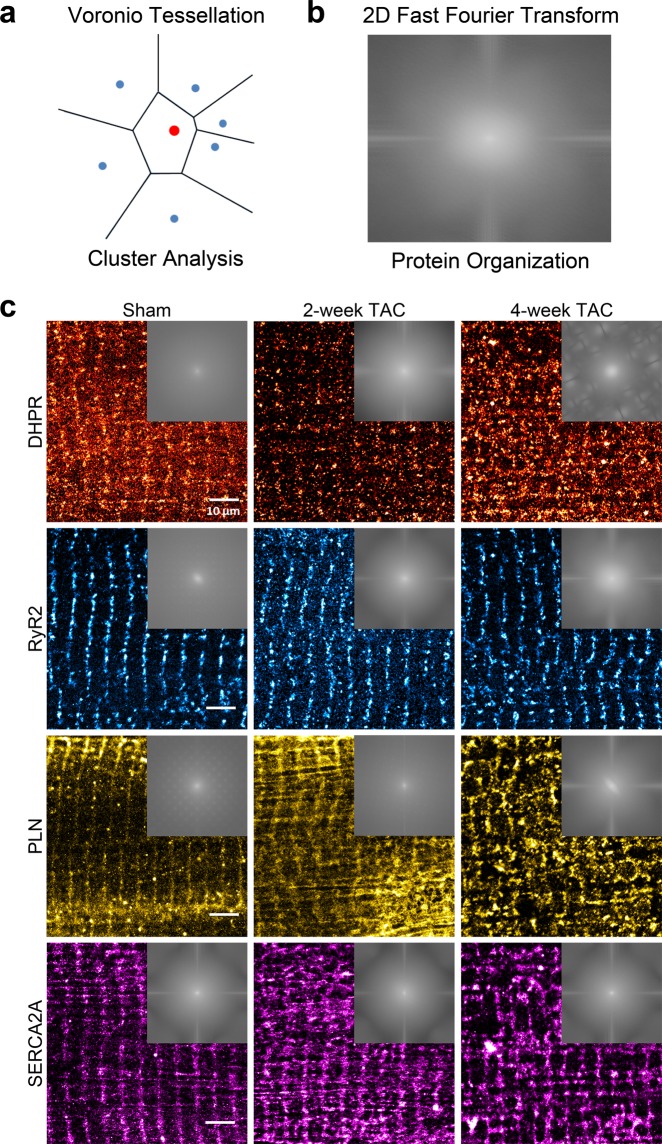
2-dimensional Fast Fourier Transformation analysis determines regional protein organization. (**a**) Schematic illustration demonstrating quantitative voronoi tessellation analysis for protein properties. (**b**,**c**) 2D FFT power spectra generated from selected regions of interests ranging between 10 × 10 to 20 × 20 μm for each protein for sham, 2-weekTAC and 4-week TAC. (**c**) Representative power spectrum is shown for each protein for sham, 2-week TAC, and 4-week TAC.

**Figure 9:**
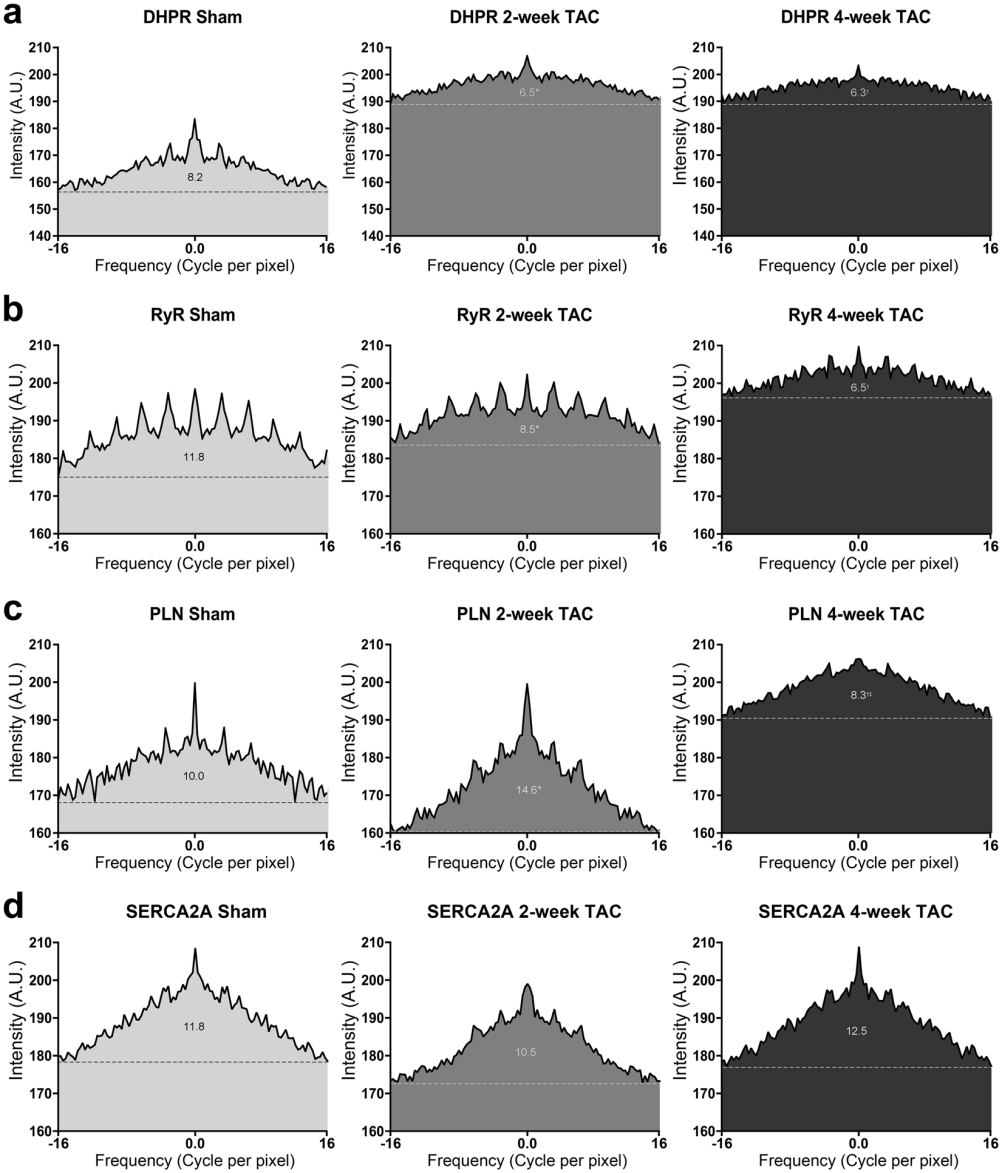
2-dimensional Fast Fourier Transformation analysis yields substantial changes in SR protein organization in pressure-overload cardiac hypertrophy. 2D-FFT analysis of two 20μmx20μm regions of interest in each cardiomyocyte was conducted for each protein in sham, 2-week TAC, and 4-week TAC and plotted for (**a**) DHPR, (**b**) RyR2, (**c**) PLN, and (**d**) SERCA2A. The dotted line in each FFT plot demonstrates the FFT signal and the area under the curve of the signal is numerically demonstrated above the dotted line. Sample size: 10–12 cells per protein per condition, n = 3; *p < 0.05 sham vs 2-week, ^†^p < 0.05 sham vs 4-week, ^‡^p < 0.05 2-week vs 4-week.

Next, we conducted 2-dimensional Fast Fourier Transform (2D-FFT) analysis on 10 regions of interest (ROIs) per protein per condition, as described in the Experimental section (Fig. 8a). For each power spectrum a rectangular selection is made over high frequency elements around the center of the spectrum and a line profile is generated; representative images in each experimental condition with their respective spectrum selection are shown in Fig. 8b. In 2D FFT, fewer, more intense peaks on the graph represent a more organized pattern of expression. Sham cardiomyocytes immunolabelled for DHPR presented a high frequency of ordered elements accompanied by a low baseline (Fig. 9a). 2-week TAC shows a reduced difference between the baseline and higher frequency elements. This was further exacerbated in 4-week TAC cardiomyocytes, demonstrating an increasingly disorganized pattern with disease progression. 2D-FFT analysis of RyR2 in sham cardiomyocytes demonstrated an ordered organizational pattern (Fig. 9b). With disease progression, we observed a higher baseline accompanied with gradually fading, more frequent and indistinct peaks, representative of less distinguishable periodicities and an overall higher disorganization.

PLN displayed increasingly smaller, high frequency peaks in 2-week and 4-week TAC cardiomyocytes (Fig. 9c). Noticeably, the baseline was lower in 2-week TAC as opposed to sham cardiomyocytes; this pattern disappeared in 4-week TAC cardiomyocytes, which exhibited a higher baseline. Analyzing the organizational differences in SERCA2A expression did not yield dramatic differences comparable to those observed in other proteins (Fig. 9d). 2-week TAC cardiomyocytes showed a reduced organization as demonstrated by the lower peaks; interestingly, however, 4-week TAC recapitulated the pattern shown in sham cardiomyocytes.

In order to quantitatively analyze the peaks identified from the FFT spectrum, we removed the baseline and performed statistical analysis of the area under the curve (AUC) of the peaks, shown above the dotted line in Fig. 9. The AUC of the signal in DHPR-stained cells decreased from 8.2 ± 0.4 in sham cardiomyocytes to 6.5 ± 0.3 and 6.3 ± 0.4 in 2-week TAC and 4-week TAC, respectively. RyR2 signal AUC also decreased progressively from 11.8 ± 0.8 in sham to 8.5 ± 0.4 in 2-week TAC and 6.5 ± 0.2 in 4-week TAC. In contrast, there was no significant change in SERCA2A’s FFT signal AUC in 2-week TAC nor 4-week TAC. Consistent with the findings of the cluster analysis, PLN had differential outcomes between 2-week and 4-week TAC. In 2-week TAC, the AUC increased significantly, from 10.0 ± 0.5 to 14.6 ± 0.6, demonstrating increased order in PLN expression; however, by 4-week TAC, it had decreased significantly to 8.3 ± 0.3 showing disorder in PLN expression with disease progression. One of the intriguing aspects of this study was that the 2D-FFT analysis of DHPR and RyR2 demonstrated such an increase in disarray during TAC. This effect was seen by the increased baseline, the lower signal peaks; both of which aligned well with the cluster analysis showing reduced density of clusters. Our data reveal a more disorganized expression with disease progression.

Interestingly, for SERCA2A, a protein mainly responsible for removing Ca^2+^ from the cytosol, 2D-FFT demonstrated an increase in the sequestration of SERCA2A with 2-week and 4-week TAC. These changes were consistent with the cluster analysis that revealed an increase in the average number of detection of SERCA2A per cluster, increased cluster area, and an increased cluster density. This increase in SERCA2A cluster area, cluster density and organization with pathological hypertrophy was mirrored by its inhibitory protein, PLN, as 2D-FFT showed increased organization and reduced number of detection and cluster area and increase in cluster density in 2-week TAC. At 4-week TAC, PLN became much more disorganized as shown by the lower peaks and higher baseline. It is interesting to note that cluster analysis shows the average number of detection, cluster area, and cluster density of 4-week TAC cardiomyocytes reverted to sham levels. The reasons behind these changes in the pattern of expression of PLN remain unclear.

### Pathological cardiac hypertrophy increases the amount of SERCA2A-containing nuclear invaginations

**Figure 10:**
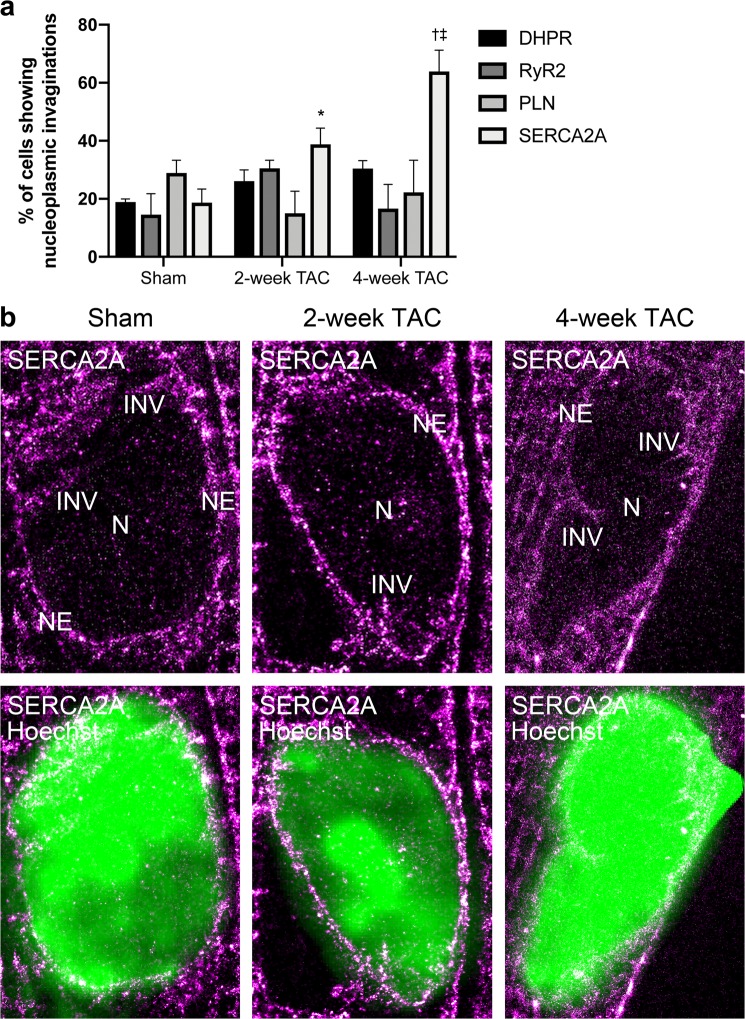
TAC cardiomyocytes present a higher frequency and abundance of SERCA2A-containing nuclear invaginations. (**a**) Cardiomyocyte nuclei imaged with dSTORM were analyzed for presence of invaginations containing target SR proteins and number of cells with nuclear invaginations for each condition were plotted. n = 3. (**b**) Representative nuclei of dSTORM-imaged SERCA2A-containing nuclear invaginations in sham, 2-week TAC, and 4-week TAC cardiomyocytes.

Interestingly, we noticed alterations in the abundance of nucleoplasmic invaginations with TAC throughout our studies. Nucleoplasmic invaginations into the nucleus have been shown to be involved in bidirectional intracellular communication and have been postulated to modulate gene expression^23–25^. In fact, regulation of gene expression is critical in the cardiomyocyte hypertrophic response^26^. We have previously shown the presence of SR protein-containing extensions of the nuclear membrane into the nucleus in cardiomyocytes, however, the purpose and development of these invaginations remain unclear in a cardiac context^27^. The high spatial resolution of dSTORM imaging used in this study provided us with a powerful tool to visualize these invaginations and attempt to shed some light on potential functions of these invaginations. We calculated the frequency of these invaginations containing each protein in sham, 2-week TAC, and 4-week TAC cardiomyocytes in our super-resolution dSTORM images in Fig. 3. The presence of DHPR-, RyR2-, and PLN-containing nuclear invaginations remained consistent in disease. However, SERCA2A-containing invaginations increased from approximately 20% of cells having an invagination to 40% of 2-week TAC and 60% of 4-week TAC cardiomyocytes (Fig. 10a). Representative changes in these SERCA2A containing invaginations have been shown in Fig. 10b.

Ca^2+^ has been shown to be an important modulator of gene transcription and expression^28–30^. Thus, the presence of Ca^2+^ regulatory proteins within these nuclear invaginations may enable local Ca^2+^ cycling modulation within discrete domains close to/within the nucleus and could therefore have a tightly coordinated ability to regulate gene expression^31^. Alterations within the composition and localization of these microdomains would be consistent with either pathological remodelling or potential compensatory responses. High resolution imaging data could provide some insight on the function of these dynamic nuclear invaginations in cardiomyocytes and their relevance in disease.

### Limitations

Despite significant changes in the patterns of expression and localization of the proteins in TAC cardiomyocytes, there are limitations linked to labeling strategy and penetration depth for nuclear invagination analysis. First, our results depend on the quality of the primary antibodies. We feel that we have used outstanding, well-used, and verified primary antibodies that have been used in the field for decades. The antibodies chosen in this study were carefully selected and have been proven to be among the most specific in the literature. As well, we have used a primary and AF647-conjugated secondary antibody labeling strategy. As multiple secondary antibodies bind to a single primary antibody, with each secondary antibody harbouring multiple fluorophores, there is some uncertainty with regards to the stoichiometry of protein labeling. Accordingly, we cannot claim that a single localization corresponds to a single protein^32^. However, despite such limitations our consistent experimental design and high sample numbers should minimize any confounding factors within this study. Furthermore, since we are imaging in total internal reflectance fluorescence (TIRF) depth, it is not unexpected that we were not able to image the nucleus in every cell image. However, despite not having imaged all the available nuclei, we still observed a significant difference between sham and TAC cardiomyocytes for SERCA2A-containing nuclear invaginations. Future studies will benefit from multi-colour super resolution experiments that can measure the spatial co-occurrence of proteins. Protein self-association states could also be explored using FRET-based methods within clustered regions to further link protein self-association states to spatial distribution. For instance, it is known that the pentameric state of PLN has a vastly different function compared to its monomeric state^33^. Finally, live-cell imaging of cardiomyocytes would provide dynamics of spatial organization changes in the progression of disease or responses to cellular stress. Given the significant advances in the field of high-resolution microscopy such approaches will soon be applied to provide significant insight into cardiomyocyte ultrastructure in health and disease.

## Conclusion

In our study we have shown quantitative differences in the 2-D nanoscale spatial distribution of critical sarcoplasmic reticulum proteins with TAC, which we feel provides a comprehensive understanding of disease progression alongside usual biochemical analyses. Most studies investigating sarcoplasmic reticulum changes in pathological cardiac hypertrophy have focussed on the biochemical changes. Very few studies have focussed on the spatial reorganization of cardiomyocyte SR in disease. To date, most studies taking advantage of super-resolution microscopy to investigate cardiac SR have focussed on healthy cells and have looked at one or two proteins^34–37^. Here, we provide quantitative evidence of SR spatial changes in pathological hypertrophy with a focus on four critical SR proteins. Voronoi tessellation cluster analysis revealed significant changes in cluster area, number of localizations per cluster, and cluster density of DHPR, RyR2, PLN, and SERCA2A with TAC. Additionally, 2-D Fast Fourier Transform analysis showed remarkable changes in the degree of spatial organization, representing the first study characterizing critical cardiac Ca^2+^ regulating proteins organization in the progression of pathological cardiac hypertrophy in nanoscale. Taken together, our study reports detailed compensatory hypertrophy-induced molecular adaptations within the cardiomyocyte. Upon pathological hypertrophy, SR proteins responsible for increasing cytosolic Ca^2+^ and sequesters SR proteins involved in removing cytosolic Ca^2+^ are significantly dispersed in the myocyte, further altering their effectiveness. Such ultrastructural alterations must be considered in future studies, in addition to alterations in biochemical analyses.

## Supplementary information

Supplementary Information
