# Supplementary material for: Nanoscale reorganization of sarcoplasmic reticulum in pressure-overload cardiac hypertrophy visualized by dSTORM
Source: Sci Rep. 2019 May 27;9:7867. doi: 10.1038/s41598-019-44331-y (PMC6536555; doi:10.1038/s41598-019-44331-y)
Supplement: Supplementary file 1 — Supplementary Information [file 41598_2019_44331_MOESM1_ESM.pdf]

Nanoscale reorganization of sarcoplasmic reticulum in pressure-overload cardiac hypertrophy visualized by dSTORM

Sina Hadipour-Lakmehsari[1,2]*, Amine Driouchi[3,4,5]*, Shin-Haw Lee[1,2], Uros Kuzmanov[1,5], Neal I. Callaghan[1,4], Scott P. Heximer[1,2], Craig A. Simmons[1,4], Christopher M. Yip[3,4,5†], Anthony O. Gramolini[1,2†]

Supplementary Information

Figure S1. Spinning-disk confocal imaging analysis of NCX1 co-stained with DHPR, RyR2, PLN, and SERCA2A in acutely isolated adult cardiomyocytes. (a) Bright-field imaging of acutely isolated adult mouse ventricular cardiomyocytes. Yellow box indicates the region of interest for analysis presented in b and c. Scale bar = 10μm. (b) Confocal imaging of NCX1 in adult cardiomyocytes displays its distinctive subcellular localization to the PM and SR. Scale bar = 10μm (left), 2μm (right). (c) Confocal imaging analysis of NCX1 co-stained with DHPR, RyR2, PLN, and SERCA2A in acutely isolated adult mouse cardiomyocytes. Yellow arrows indicate regions of co-localization. Scale bar = 2μm.

Figure S2. Confocal imaging analysis of isolated adult mouse cardiomyocytes stained with Alexa Fluor-647 secondary antibodies as an internal control for immunofluorescence experiments. Scale bar = 20μm.

Figure S3. Original uncropped western blots.

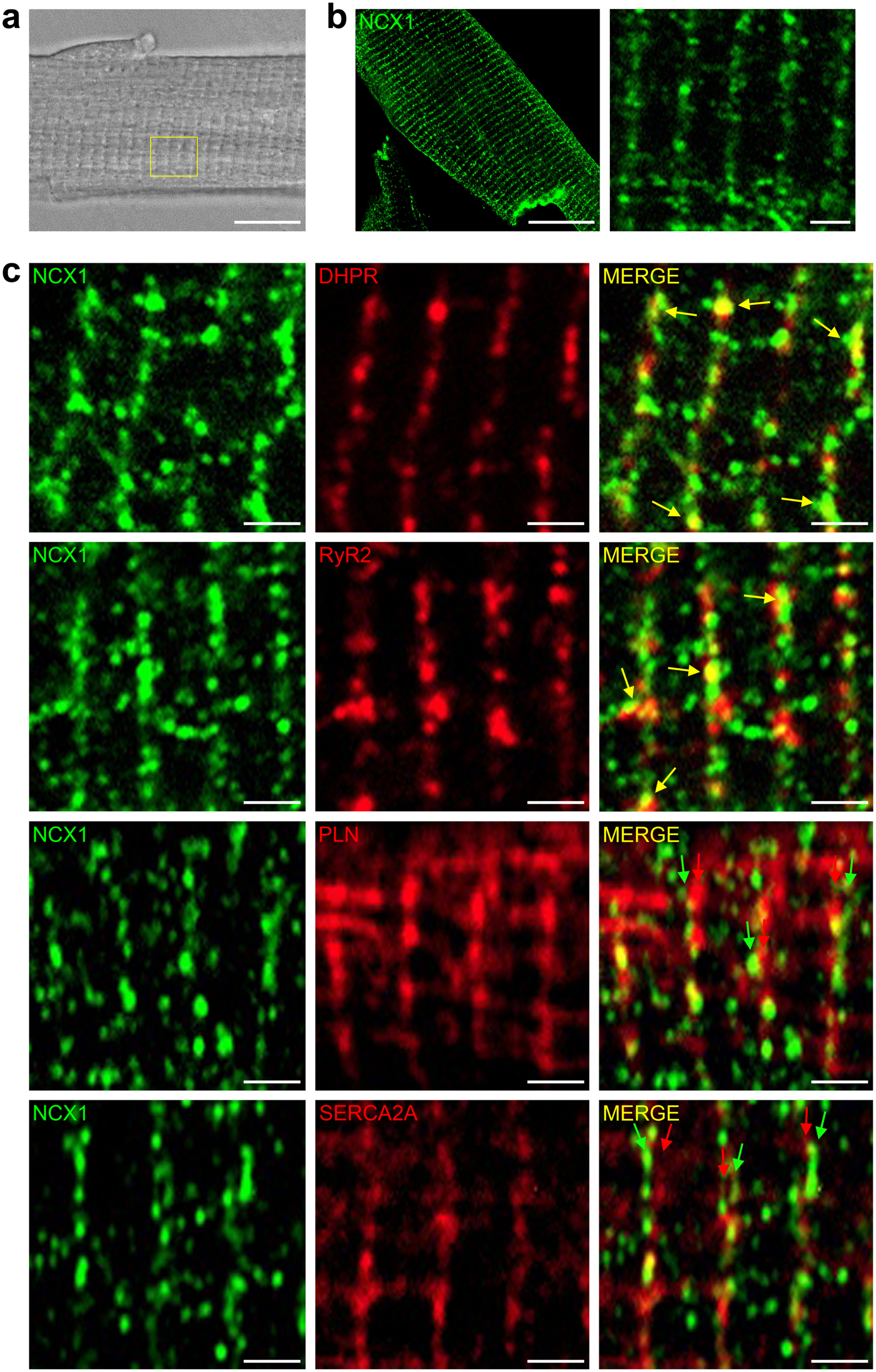
a
b
NCX1
c
NCX1
DHPR
MERGE
NCX1
RyR2
MERGE
NCX1
PLN
MERGE
NCX1
SERCA2A
MERGE

a Confocal Imaging of isolated adult mouse cardiomyocytes stained with Alexa647 only

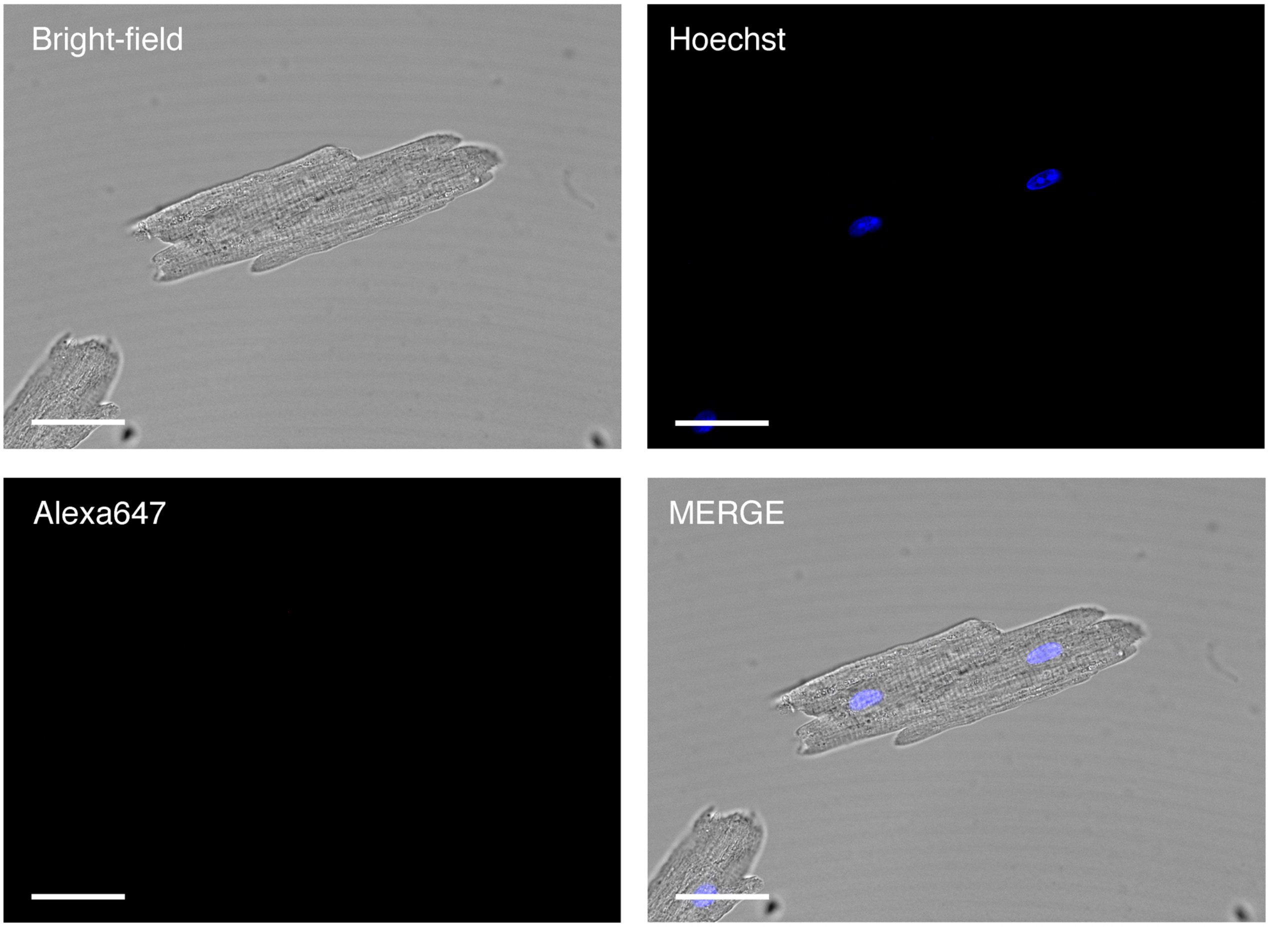

Fig. 1b

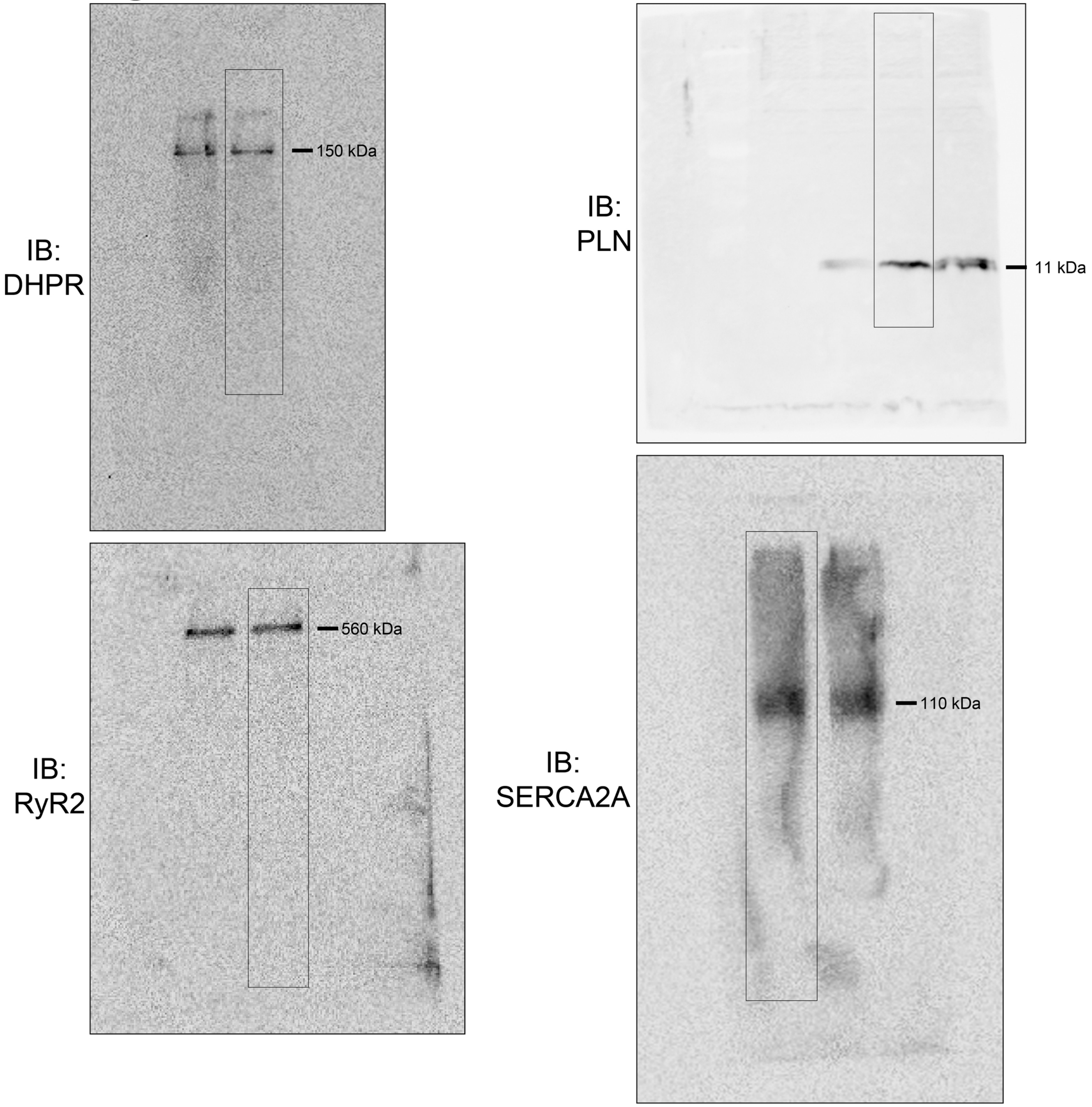

Fig. 5a

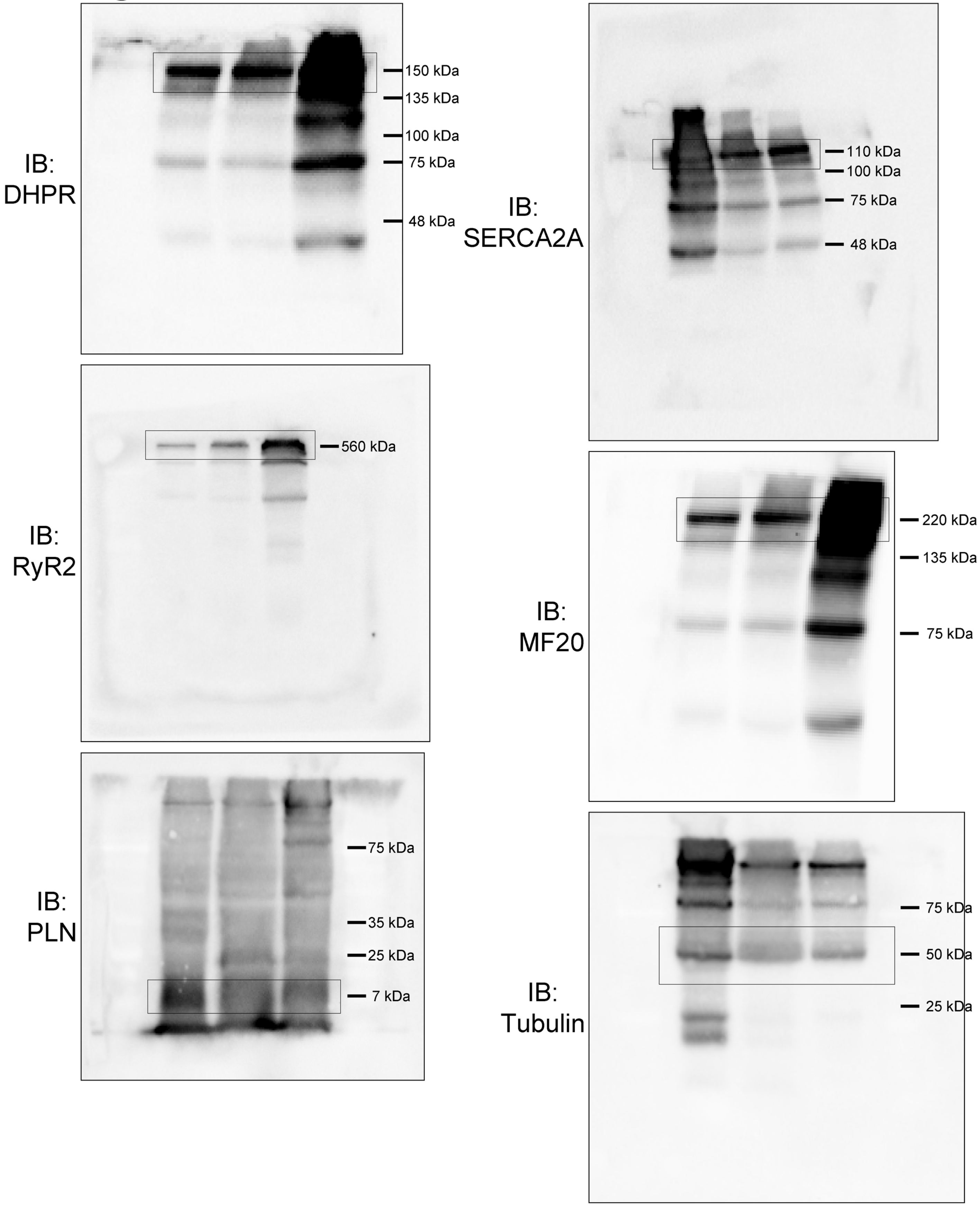
IB: DHPR
150 kDa
135 kDa
100 kDa
75 kDa
48 kDa
IB: SERCA2A
110 kDa
100 kDa
75 kDa
48 kDa
IB: RyR2
560 kDa
IB: MF20
220 kDa
135 kDa
75 kDa
IB: PLN
75 kDa
35 kDa
25 kDa
7 kDa
IB: Tubulin
75 kDa
50 kDa
25 kDa
